# Negative modulation of suppressive HIV-specific regulatory T cells by IL-2 adjuvanted therapeutic vaccine

**DOI:** 10.1371/journal.ppat.1006489

**Published:** 2017-07-14

**Authors:** Vedran Brezar, Lylia Hani, Mathieu Surenaud, Audrey Hubert, Christine Lacabaratz, Jean-Daniel Lelièvre, Yves Levy, Nabila Seddiki

**Affiliations:** 1 Inserm, U955, Equipe 16, Créteil, Paris, France; 2 Université Paris Est, Faculté de médecine, Créteil, Paris, France; 3 Vaccine Research Institute (VRI), Créteil, Paris, France; 4 AP-HP, Hôpital H. Mondor - A. Chenevier, Service d'immunologie clinique et maladies infectieuses, Créteil, Paris, France; Emory University, UNITED STATES

## Abstract

The potential benefit in using IL-2 in immunotherapy for cancer and autoimmunity has been linked to the modulation of immune responses, which partly relies on a direct effect on Tregs populations. Here, we revisited the role of IL-2 in HIV infection and investigated whether its use as an adjuvant with therapeutic vaccination, impacts on HIV-specific responses. Antiretroviral therapy treated-patients were randomized to receive 4 boosts of vaccination (ALVACHIV/Lipo-6T, weeks 0/4/8/12) followed by 3 cycles of IL-2 (weeks 16/24/32) before treatment interruption (TI) at week40. IL-2 administration increased significantly HIV-specific CD4^+^CD25^+^CD134^+^ T-cell responses, which inversely correlated with viral load after TI (r = -0.7, p <0.007) in the vaccine/IL-2 group. IL-2 increased global CD25^+^CD127^low^FoxP3^+^Tregs (p <0.05) while it decreased HIV- but not CMV- specific CD39^+^FoxP3^+^CD25^+^CD134^+^Tregs (p <0.05). HIV-specific Tregs were inversely correlated with IFN-γ producing specific-effectors (p = 0.03) and positively correlated with viral load (r = 0.7, p = 0.01), revealing their undesired presence during chronic infection. Global Tregs, but not HIV-specific Tregs, inversely correlated with a decrease in exhausted PD1^+^CD95^+^ T-cells (p = 0.001). Altogether, our results underline the negative impact of HIV-specific Tregs on HIV-specific effectors and reveal the beneficial use of IL-2 as an adjuvant as its administration increases global Tregs that impact on T-cell exhaustion and decreases HIV-specific CD39^+^Tregs by shifting the balance towards effectors.

## Author summary

Interleukin-2 (IL-2) has been used in immunotherapy for cancer and autoimmunity and its beneficial effect has been linked to the modulation of immune responses, which partly relies on a direct effect on Tregs populations. In this study, we assessed the role of IL-2 in HIV infection and investigated whether its use as an adjuvant with therapeutic vaccination, impacts on HIV-specific responses. We show that IL-2 administration increased HIV-specific CD4^+^CD25^+^CD134^+^ T-cell responses which inversely correlated with viral load after treatment interruption in the vaccine/IL-2 group. We also show that IL-2 increased global CD25^+^CD127^low^FoxP3^+^Tregs while it decreased HIV- but not CMV- specific CD39^+^FoxP3^+^CD25^+^CD134^+^Tregs. Moreover, we show that HIV-specific Tregs were inversely correlated with IFN-γ-producing specific-effectors and positively correlated with viral load. Moreover, we show that global Tregs, but not HIV-specific Tregs, inversely correlated with a decrease in exhausted PD1^+^CD95^+^ T-cells. Altogether, our results underline the negative impact of HIV-specific Tregs on HIV-specific effectors and reveal the beneficial use of IL-2 as an adjuvant as its administration increases global Tregs that impact on T-cell exhaustion and decreases HIV-specific CD39^+^Tregs by shifting the balance towards effectors.

## Introduction

CD4^+^ regulatory T cells (Tregs) are central in maintaining peripheral tolerance and constitute the most important extrinsic inhibitory mechanism that control T-cell responses (reviewed in [1]). Human peripheral thymic-derived naive and effector Tregs are delineated as CD4^+^CD25^hi^CD127^low^FoxP3^+^ CD45RA^+^ and CD45RA^-^ respectively [2–4], while circulating antigen-specific Tregs, best at regulating targeted immune responses, can be identified by the expression of co-stimulatory molecules such as CD134 (OX40) [5,6] or CD137 (4-1BB) [7,8]. We have recently shown in a therapeutic vaccine study, that vaccinees who displayed lower levels of HIV-specific CD4^+^CD134^+^CD25^+^CD39^+^FoxP3^+^ Tregs showed better responses to the vaccine even though global CD4^+^CD25^hi^CD127^low^FoxP3^+^ were slightly increased, probably reflecting restoration of CD4^+^ T-cell compartment [9]. However, Tregs subsets dynamics and the particular role played by each subset during chronic infection are still unclear.

Targeting Tregs subsets to shift the balance between tolerance and immunity in the clinic remains challenging. To this end, the use of recombinant interleukin 2 (rIL-2) has been beneficial as several successes of low-dose rIL-2 therapy in animal models of autoimmune pathology [10–14] and human clinical studies in hepatitis C virus induced vasculitis, chronic graft-versus-host disease (GVHD), Type1 Diabetes (T1D), systemic lupus erythematosus (SLE), and Alopecia areata [15–21] have been reported. These successes led to other clinical trial studies, including rheumatoid arthritis, ankylosing spondylitis, psoriasis, Behcet’s disease, Crohn’s disease and ulcerative colitis (TRANSREG, clinicalTrials.gov NCT01988506). The advantageous function of low-dose rIL-2 in this context has been linked to the expansion of Tregs that play a major role in controlling immune responses and establishing tolerance [22].

In cancer immunotherapy, high-dose intermittent rIL-2 therapy has increased long-term survival for some patients with metastatic renal cell carcinoma [23] and rIL-2 therapy alone or in combination with a peptide vaccine has resulted in clinical improvement for patients with metastatic melanoma [24,25]. However, the use of rIL-2 to enhance immune restoration in infectious diseases such as in human immunodeficiency virus (HIV-1) infection did not have similar success. Indeed, two major clinical trial studies, SILCAAT and ESPRIT, have been conducted to expand the CD4^+^ T-cell pool in HIV-1-infected patients and despite a substantial and sustained increase in the CD4 count as compared with antiretroviral therapy (HAART) alone, rIL-2 plus HAART yielded no clinical benefit [26]. These disappointing results were explained by the expansion of two distinct CD4^+^CD25^+^ T-cell populations CD4^+^CD25^low^CD127^low^FoxP3^+^ and CD4^+^CD25^hi^CD127^low^FoxP3^hi^ with gene expression profiles similar to those of CD4^+^CD25^hi^FoxP3^+^ Tregs [27]. However, when continuous IL-2 administration has been used as an adjuvant along with therapeutic vaccination and antiretroviral treatment, in simian immunodeficiency virus (SIV) infected macaques, the results showed increased SIV-specific CD8 T-cell responses and resulted in decreased viral load (VL) [28,29]. In humans, greater improvement was seen in one trial (ANRS 093), where patients were randomized to continue either HAART alone or to receive therapeutic vaccines (ALVAC-HIV and Lipo-6T) followed by 3 cycles of subcutaneous IL-2 [30,31]. The results showed that therapeutic immunization combining ALVAC-HIV and Lipo-6T vaccines followed by IL-2 administration, induced sustained and broad CD4 T-cell immune responses to HIV antigens in chronically HIV-infected patients, that correlated with a partial control of viral replication following treatment interruption (TI) [30,31].

In this ANRS 093 follow-up study, we aimed to comprehensively analyse changes in the phenotype and function of Tregs subsets and hypothesized that by using IL-2 as an adjuvant along with therapeutic vaccination, we may affect distinctively peripheral global CD4^+^ CD25^hi^ CD127^low^ FoxP3^+^ and HIV-specific CD4^+^ CD134^+^ CD25^+^ CD39^+^ FoxP3^+^ Tregs. We show that therapeutic vaccine associated to IL-2 adjuvant increases global Tregs that impacts on T-cell exhaustion and decreases HIV-specific Tregs thus shifting the balance towards effectors.

## Results

### Therapeutic immunization strategy

**Fig 1:**
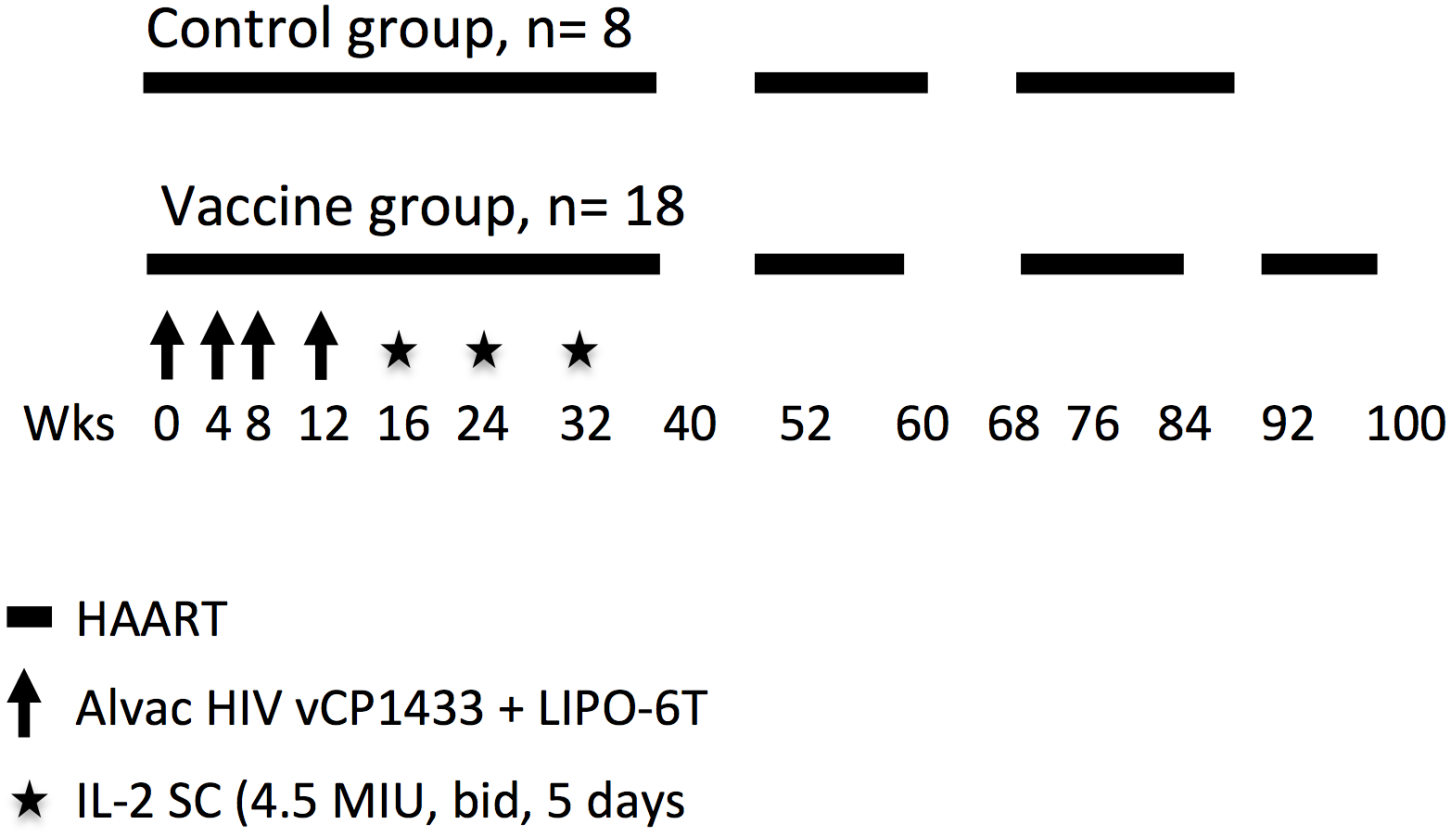
Therapeutic immunization strategy. HAART treated chronic HIV-infected subjects received two vaccines: recombinant ALVAC–HIV (vCP1433) and Lipo-6T (HIV-1 lipopeptides), followed by administration of three cycles of subcutaneous IL-2. Patients received a schedule of four shots of vaccine or placebo, every four weeks followed by three cycles of IL-2 at 4.5MIU (two injections a day for five days). Eight weeks after the last IL-2 cycle, HAART was stopped and patients’ viral load measured. The treatment was reintroduced only if viral load was over 50,000 copies/ml at one measurement or over 10,000 copies/ml at two consecutive measurements.

This clinical trial is part of the ANRS 093 randomized study. As detailed in the methods section and summarized in Fig 1, HAART treated chronic HIV-infected subjects received two different vaccines: four shots of recombinant ALVAC–HIV (vCP1433) and HIV LIPO-6T (HIV-1 lipopeptides + TT) or placebo every four weeks, followed by administration of three cycles of subcutaneous IL-2 at 4.5MIU (two injections a day for five days). Cells from 26 patients (n = 8 placebo and n = 18 vaccine/IL2) were available amongst the 70 patients included in the ANRS 093 trial. In this study, we focused our analyses on vaccine/IL-2 group patients (n = 18) before (wk0) and after vaccine (wk16) and IL-2 treatment (wk36). Placebo group has not been included in our analyses, apart from Table 1 where vaccine/IL-2 group characteristics were compared to the placebo group. As summarized in the table, patients from both groups showed no difference in CD8 and CD4 cell counts and in nadir CD4 at randomization. Plasma HIV-1 VL (copies RNA/ml) before HAART was higher in the vaccine group. After vaccination (wk36), all but 1 in placebo group had undetectable plasma HIV-1 VL and CD8 cell count was lower in vaccine group. After treatment interruption (wk40), HIV-1 RNA peak was significantly lower in the vaccine group, and a trend towards a longer time to viral peak rebound was observed in the vaccine group as compared to the placebo group (Table 1).

**Table 1:** Patients’ characteristics.

	Placebo (n = 8)	VAC-IL2 (n = 18)	P value ^a^
Sexe	5M, 3F	14M, 4F	
Median age (years) [IQR]	38 [35–39]	41 [35–45]	
HIV RNA pre HAART (copies/ml) [IQR]	9850 [919–19243]	14658 [7185–59745]	**0.0017**
HIV RNA <50 copies/ml at baseline (No/total No)	8/8	17/18	
Median CD8 cell count at baseline (/mm^3^) [IQR]	885 [615–1168]	731 [514–964]	0.146
Median CD4 cell count at baseline (/mm^3^) [IQR]	944 [658–1095]	638 [511–778]	0.458
Median Nadir CD4 [IQR]	303 [197–318]	249 [208–286]	0.749
HIV RNA <50 copies/ml at W36 (No/total No)	7/8	18/18	
Median CD8 cell count at W36 (/mm^3^) [IQR]	1015 [790–1130]	898 [686–1137]	**0.016**
Median CD4 cell count at W36 (/mm^3^) [IQR]	1032 [815–1221]	1145 [721–1313]	0.96
Stop HAART at W40 (No/total No)	7/8	18/18	
virological success at W52(No/total No)^b^	1/7	8/18	
Median time to virological failure (days) [IQR]	29 [28–53]	72 [28–86]	0.652
Median peak HIV RNA (copies/ml) [IQR]	183000 [50250–698090]	40250 [18904–140930]	**<0.001**
Median time to peak HIV RNA (days) [IQR]	28 [24–39]	42 [28–56]	0.07

IQR, interquartile range; Vac-IL-2, ALVAC-HIV (vCP1433) and Lipo-6T vaccines plus interleukin-2

^a^Fisher test, Wilcoxon rank-sum test, log-rank test for the time variables (one-sided tests).

^b^Patients were considered as in virological success if they stopped antiviral drugs at week 40 and remained off therapy and with plasma HIV RNA values <50.000 copies/ml at week 44 and <10.000 copies/ml at week 48 thereafter until week 52

### Subcutaneous injections of IL-2 expand peripheral CD4^+^CD25^+^CD127^low^FoxP3^+^ naïve and total memory Tregs

**Fig 2:**
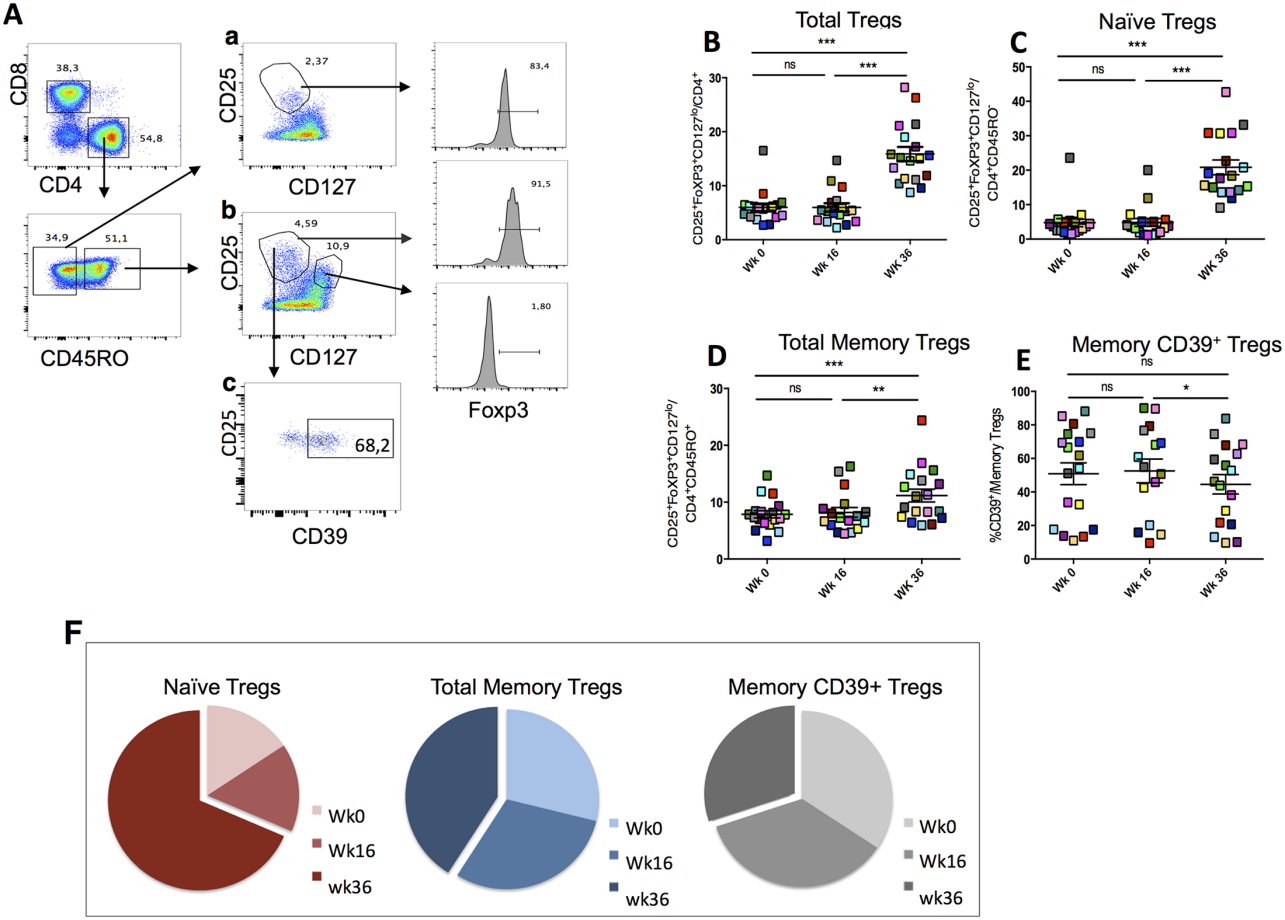
Subcutaneous injections of IL-2 expand peripheral CD4^+^CD25^+^CD127^low^Foxp3^+^ naïve and total memory Tregs. (A) Gating strategy for naïve CD4^+^ CD45RO^-^ CD25^+^ CD127^low^ FoxP3^+^, total memory CD4^+^ CD45RO^+^ CD25^+^ CD127^low^ FoxP3^+^ and memory CD4^+^ CD45RO^+^ CD25^+^ CD127^low^ FoxP3^+^ CD39^+^. (B) Proportions of total CD4^+^CD25^+^CD127^low^Foxp3^+^ Tregs, (C) naïve CD45RO^-^CD4^+^CD25^+^CD127^low^Foxp3^+^ Tregs, (D) total memory CD45RO^+^CD4^+^CD25^+^CD127^low^Foxp3^+^ Tregs and (E) memory CD39^+^Tregs, were measured in eighteen patients at wk0, wk16 and wk36 after vaccination and IL-2 injections. Each patient can be distinguished by a colored symbol in the figures. (F) Pie chart showing comparison for compartments of Tregs subsets expansion at wk0, wk16 and wk36. Prism 5.0, version 5.0d, (GraphPad Software, Inc.) was used for statistical analyses. P values were considered significant when < 0.05.

In order to assess the impact of vaccination and IL-2 therapy on Tregs expansion, we measured the frequency of three subsets of Tregs in patients at wk0, wk16 and wk36, namely: naïve CD4^+^ CD45RO^-^ CD25^+^ CD127^low^ FoxP3^+^, total memory CD4^+^ CD45RO^+^ CD25^+^ CD127^low^ FoxP3^+^ and memory CD4^+^ CD45RO^+^ CD25^+^ CD127^low^ FoxP3^+^ CD39^+^ as shown in the gating strategy (Fig 2A). The 3 subsets have been identified and their proportions measured in eighteen patients (Table 1). Each patient can be distinguished in the figures by a colored symbol.

The results show that while no changes were observed between wk0 and wk16 (Fig 2), proportions of total Tregs and among them both naïve CD45RO^-^ CD25^+^CD127^low^FoxP3^+^ and total memory CD45RO^+^ CD25^+^CD127^low^FoxP3^+^ Tregs, significantly increased at wk36 compared to wk0 (mean ±SEM for naive Tregs: 4.8% ±1.1 *vs* 20.8% ±2.1, p<0.0001 and total memory Tregs: 8.2% ±0.8 *vs* 11.2% ±1.1, p<0.01; (panel C and D, Fig 2)), demonstrating the direct effect of IL-2 on both IL-2 receptor α-chain-expressing cell subsets (Fig 2B–2D). The only exception was the memory CD39^+^ subset among total memory Tregs that decreased after IL-2 administration (mean ±SEM of 52.5% ±7.06 *vs* 44.5% ±5.7, p<0.05; Fig 2E). The comparison in Tregs expansion at wk36 after IL-2 treatment highlights the significant expansion of the naïve Tregs compartment compared to the others (Fig 2F).

### Subcutaneous injections of IL-2 decrease PD-1 expression in CD4+ and CD8^+^ T cells

Given that T-cell activation and exhaustion are two major features of chronic viral infections [32], we assessed these two characteristics using patients’ CD4^+^ and CD8^+^ T-cells at wk0, wk16 and w36. The expression of HLA-DR and CD38 molecules together with inhibitory receptors PD-1, Tim-3, 2B4 and Blimp-1 that have been shown to play a central role in inhibiting T-cell function during chronic infections, were measured [33].

**Fig 3:**
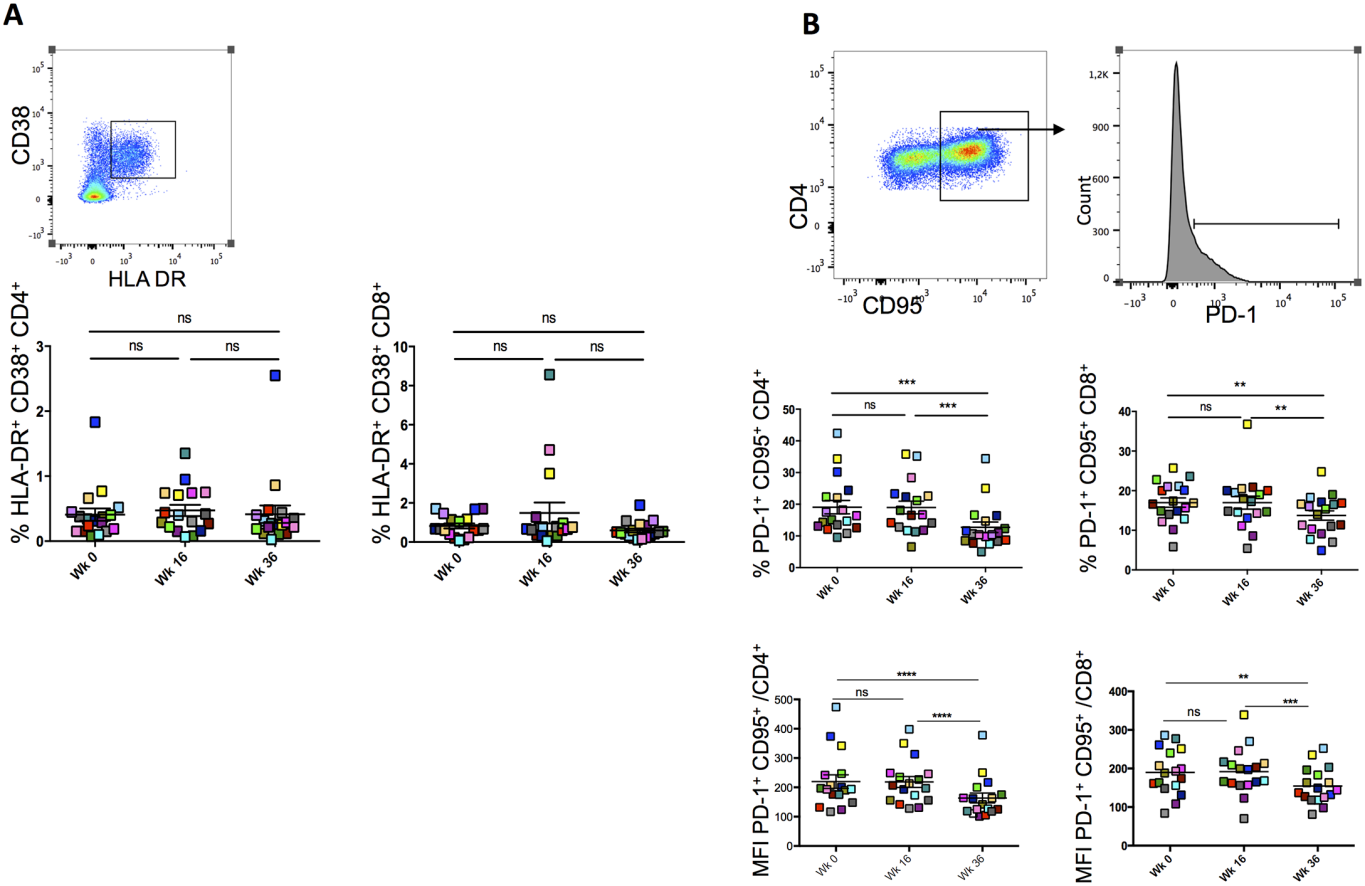
Subcutaneous injections of IL-2 decrease PD-1 expression in CD4+ and CD8+ T cells. (A) Gating strategy for CD4^+^ HLA-DR^+^CD38^+^ and CD8^+^ HLA-DR^+^CD38^+^ cell frequencies measured at wk0, wk16 and wk36. Cells were first gated on CD3+CD4+ before depicting CD38+HLA-DR+ subset. (B) Gating strategy for CD4^+^CD95^+^PD-1^+^ and CD8^+^CD95^+^PD-1^+^ cell frequencies that were measured at wk0, wk16 and wk36. CD3^+^CD4^+^ cells were first selected, then CD95^+^ cell gate applied and finally PD-1+ cells were depicted. Bottom panel shows Mean Fluorescence Intensity (MFI) for PD-1 on CD95+CD4+ and CD95^+^CD8^+^. Prism 5.0, version 5.0d, (GraphPad Software, Inc.) was used for statistical analyses. P values were considered significant when < 0.05.

**Fig 4:**
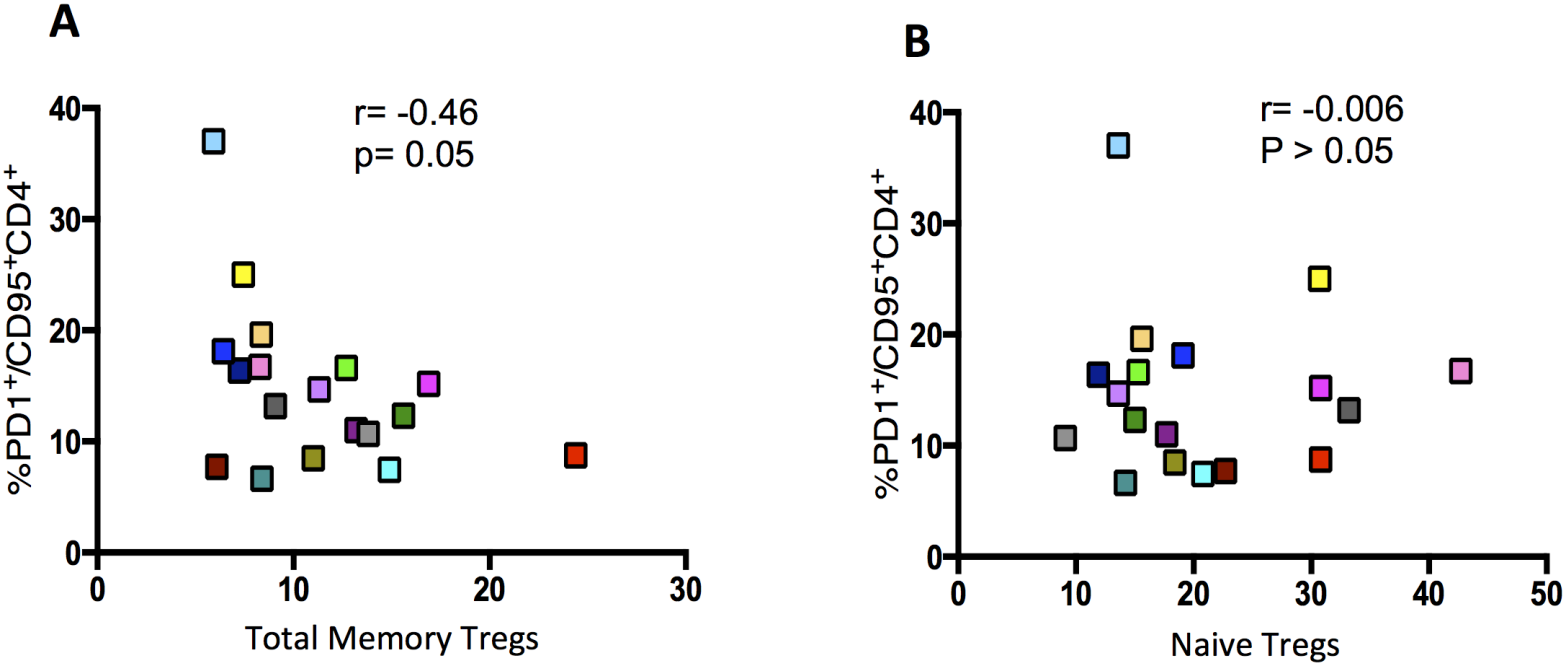
Correlation between total memory or naïve Tregs with PD1^+^ CD95^+^ cells. At week36% total memory CD45RO^+^CD25^+^CD127^low^FoxP3^+^ (A) or naïve CD45RO^-^CD25^+^CD127^low^FoxP3^+^ (B) were measured by flow cytometry and then correlated with %PD-1^+^CD95^+^CD4^+^. Correlations were calculated using spearman correlation coefficients (Prism 5.0, version 5.0d). P values were considered significant when < 0.05.

CD4^+^ and CD8^+^ HLA-DR^+^CD38^+^ cell frequencies were not affected by neither vaccine nor IL-2 administration as there were no changes in their frequencies at the three time points (p>0.05, Fig 3A) IL-2 treatment led to a significant decrease in CD4^+^CD95^+^PD-1^+^ and CD8^+^CD95^+^PD-1^+^ frequencies (19% ±2 *vs* 12.7% ±1.6, p<0.0001 and 17.1% ±1.6 vs 13.7% ±1.1 in CD4 and CD8 subsets at wk16 and wk36 respectively, p<0.001; Fig 3B). Similar results were observed with PD-1 mean fluorescence intensity (MFI) on both CD4^+^CD95^+^ and CD8^+^CD95^+^ T cells (Fig 3C). Tim-3 and Blimp-1 MFI followed similar trends (p<0.05; S1 Fig). Moreover, we observed a decrease in CD38 and HLA-DR MFI at wk36 on CD4^+^CD95^+^ cells (S1 Fig). Of note there was an inverse correlation, although not significant, between total memory CD45RO^+^CD25^+^CD127^low^FoxP3^+^ (p = 0.05) but not naïve CD45RO^-^CD25^+^CD127^low^FoxP3^+^ Tregs (p>0.05) with CD95^+^PD1^+^ CD4^+^ T cell frequencies at wk36 (Fig 4A and 4B) suggesting that these Tregs may impact on T-cell exhaustion. However, further experiments are needed to assess the role of CD45RO^+^CD25^+^CD127^low^FoxP3^+^ Tregs in the control of T-cell exhaustion.

### HIV-specific CD4^+^ T-cell responses were amplified by IL-2 treatment and inversely correlated with VL after treatment interruption

**Fig 5:**
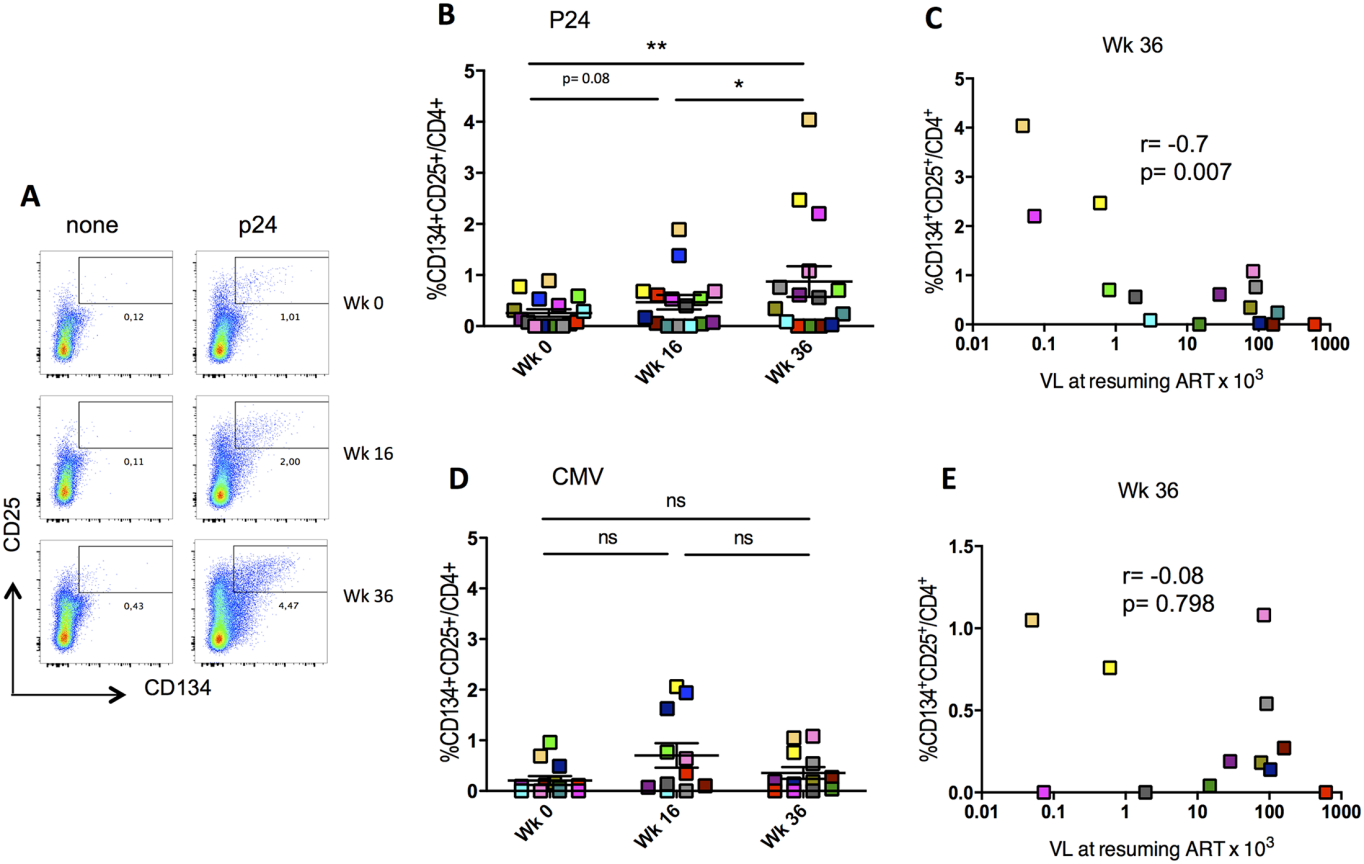
HIV-specific CD4^+^ T-cell responses were amplified by IL-2 treatment and inversely correlate with VL after treatment interruption. (A) Example of gating strategy used for detecting antigen-specific CD4^+^CD25^+^CD134^+^ cells after in vitro stimulation with p24 or none (OX40 assay), at wk0, wk16 and wk36. (B and D) Representation of HIV- and CMV- specific CD4^+^CD25^+^CD134^+^ percentages in patients at wk0, wk16 and wk36 using the gating strategy shown in (A). (C and E) Correlations between %CD4^+^CD25^+^CD134^+^ and viral load at resuming ART, at wk36 for p24 and CMV, respectively. Correlations were calculated using spearman correlation coefficients (Prism 5.0, version 5.0d). P values were considered significant when < 0.05.

We have recently shown that CD4^+^CD25^+^CD134^+^ HIV-specific responses induced after vaccination, inversely correlated with viral load rebound after TI [9]. To investigate whether the same applied in ANRS 093 clinical trial where a different vaccine was given, we measured CD4^+^ Gag-specific responses at wk0, wk16 and wk36 using the “OX40 assay” (reviewed in [1]). We observed a significant increase in HIV-specific responses only after IL-2 treatment (wk36) (wk16 vs wk36 p<0.02 and wk0 vs wk36 p<0.008, Fig 5B). This induction was antigen-dependent as only HIV-specific but not CMV-specific responses were affected (Fig 5B and 5D). In line with our previous findings, the HIV-specific responses (at wk36) inversely correlated with viral load after treatment interruption (r = -0.7 and p<0.007; Fig 5C), but this was not the case for CMV-specific responses, which did not show any correlation with HIV viral load after treatment interruption (Fig 5E). Of note, we did not see any changes in CD4^+^CD25^+^CD134^+^ HIV-specific responses in HIV-infected patients placebo group who did not receive the vaccine and IL-2 therapy.

### Negative modulation of HIV-specific Tregs by IL-2 therapy

**Fig 6:**
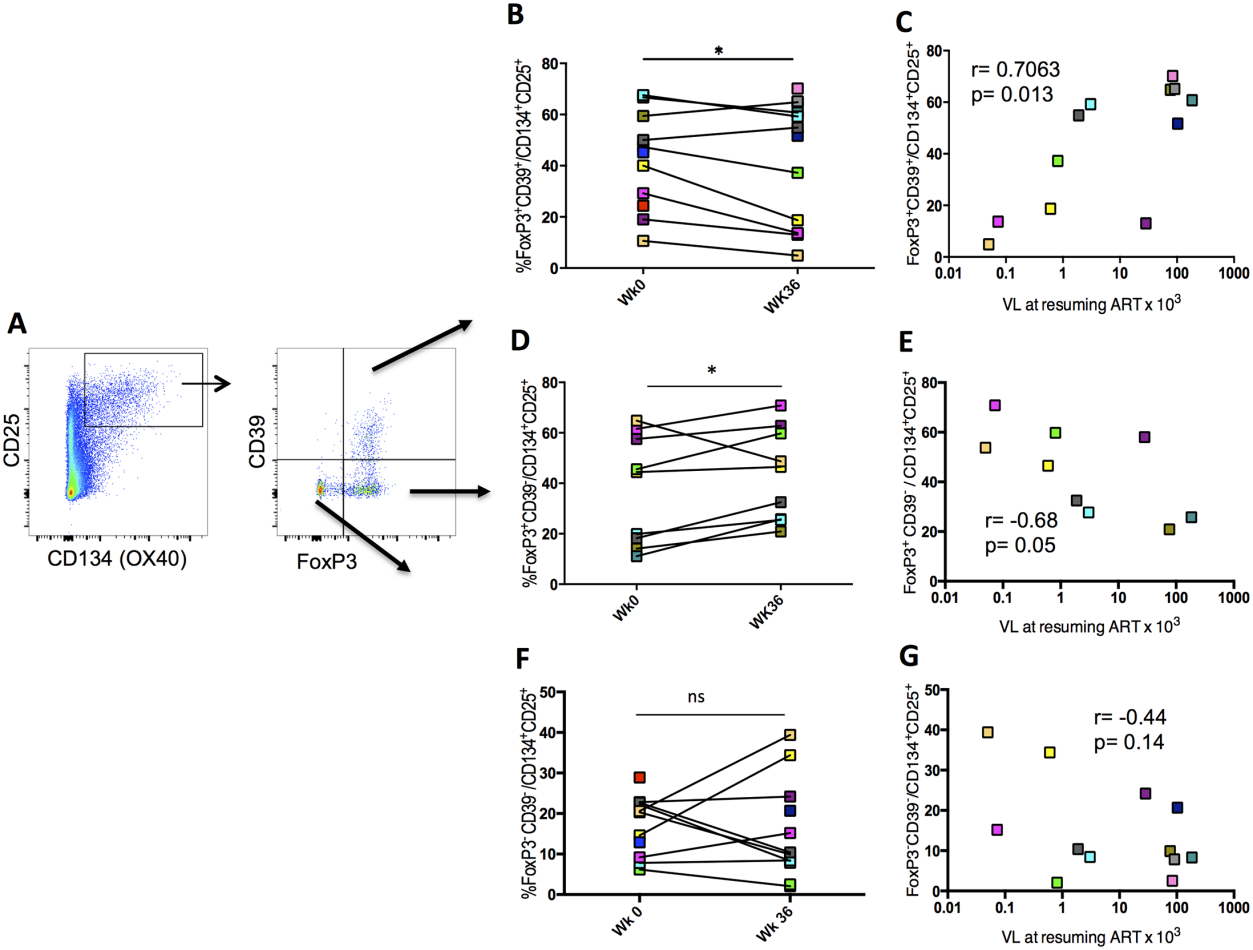
HIV-specific Tregs decrease after IL-2 treatment and positively correlate with viral load after treatment interruption. (A) Gating strategy used for detecting HIV-specific CD4^+^CD25^+^CD134^+^CD39^+^FoxP3^+^ Tregs after p24 stimulation using the “OX40 assay”. (B, D and F) HIV-specific CD4^+^CD25^+^CD134^+^CD39^+^FoxP3^+^ Tregs percentages in patients at wk0 and wk36 using the gating strategy shown in (A). (C, E and G) Correlations between %CD4^+^CD25^+^CD134^+^CD39^+^FoxP3^+^ Tregs at wk36 and viral load at resuming ART. Correlations were calculated using spearman correlation coefficients (Prism 5.0, version 5.0d). P values were considered significant when < 0.05.

**Fig 7:**
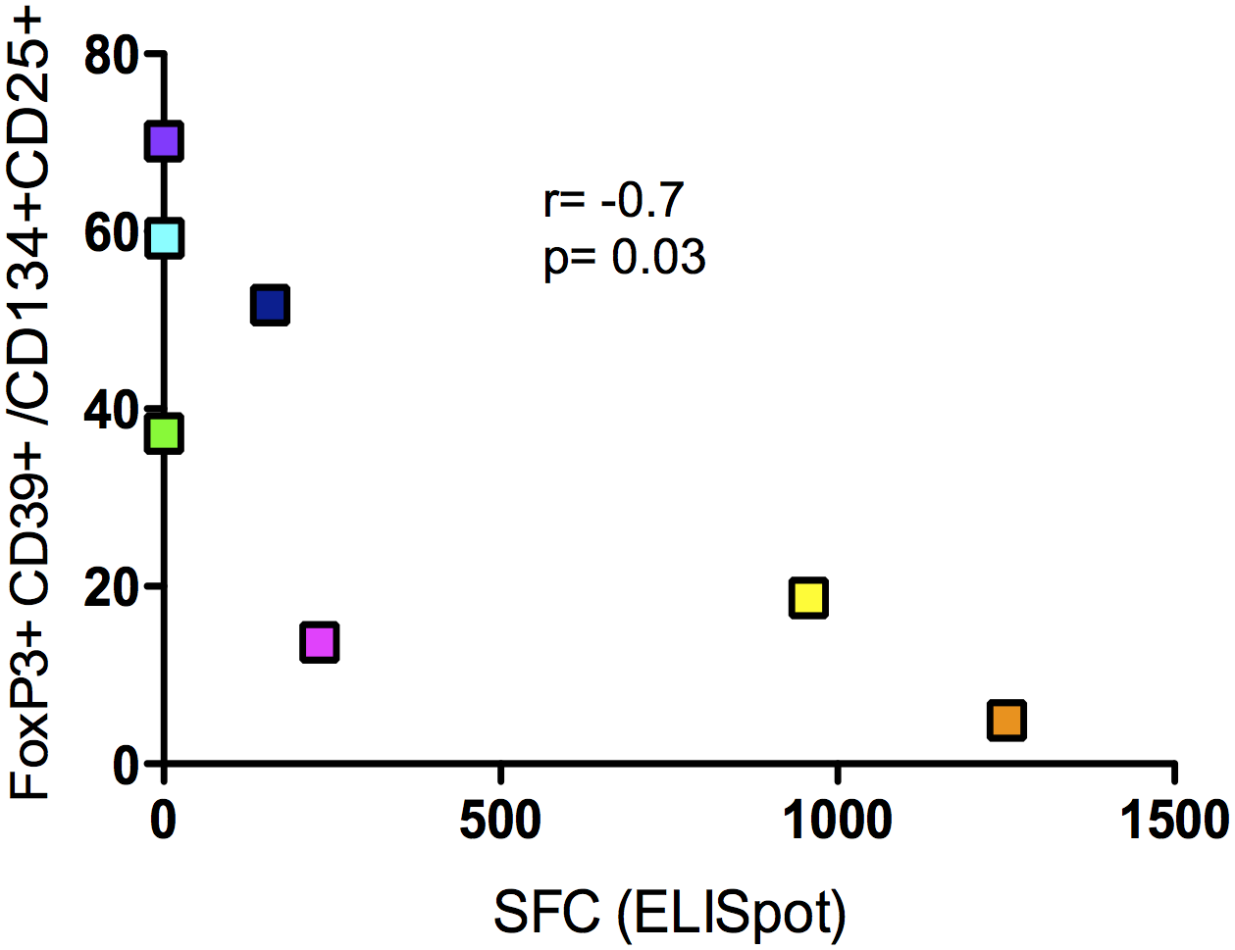
Correlations between %CD4^+^CD25^+^CD134^+^CD39^+^FoxP3^+^ Tregs and p24 IFNγ-ELISpot at wk36. IFN-γ producing cells (SFC) were measured by ELISpot as described in the methods. Correlations were calculated using spearman correlation coefficients (Prism 5.0, version 5.0d). P values were considered significant when < 0.05.

Results described above showed that IL-2 therapy down-modulated a population of Tregs expressing CD39^+^. We have previously shown that by using CD39 and FoxP3 we were able to delineate two populations of antigen-specific CD4^+^CD25^+^CD134^+^ T cells with different origin and function, namely Tregs and Teffs (Fig 6A) and [6,9] and that CD39^+^ Tregs are potent suppressors of HIV-specific responses in both natural infection and vaccination [9,34]. Accordingly, we confirm here that frequency of CD39^+^FoxP3^+^CD25^+^CD134^+^ HIV-specific Tregs was positively correlated with VL after TI (r = 0.7 and p = 0.01; Fig 6C) and interestingly IL-2 therapy decreased significantly the frequency of CD39^+^FoxP3^+^CD25^+^CD134^+^ HIV-specific Tregs, but not CMV-specific Tregs (p = 0.01, Fig 6B). We also found that frequency of CD39^+^FoxP3^+^CD25^+^CD134^+^ HIV-specific Tregs was inversely correlated with IFN-γ producing effector specific cells that were measured by ELISpot (r = -0.7, p = 0.03; Fig 7)

**Fig 8:**
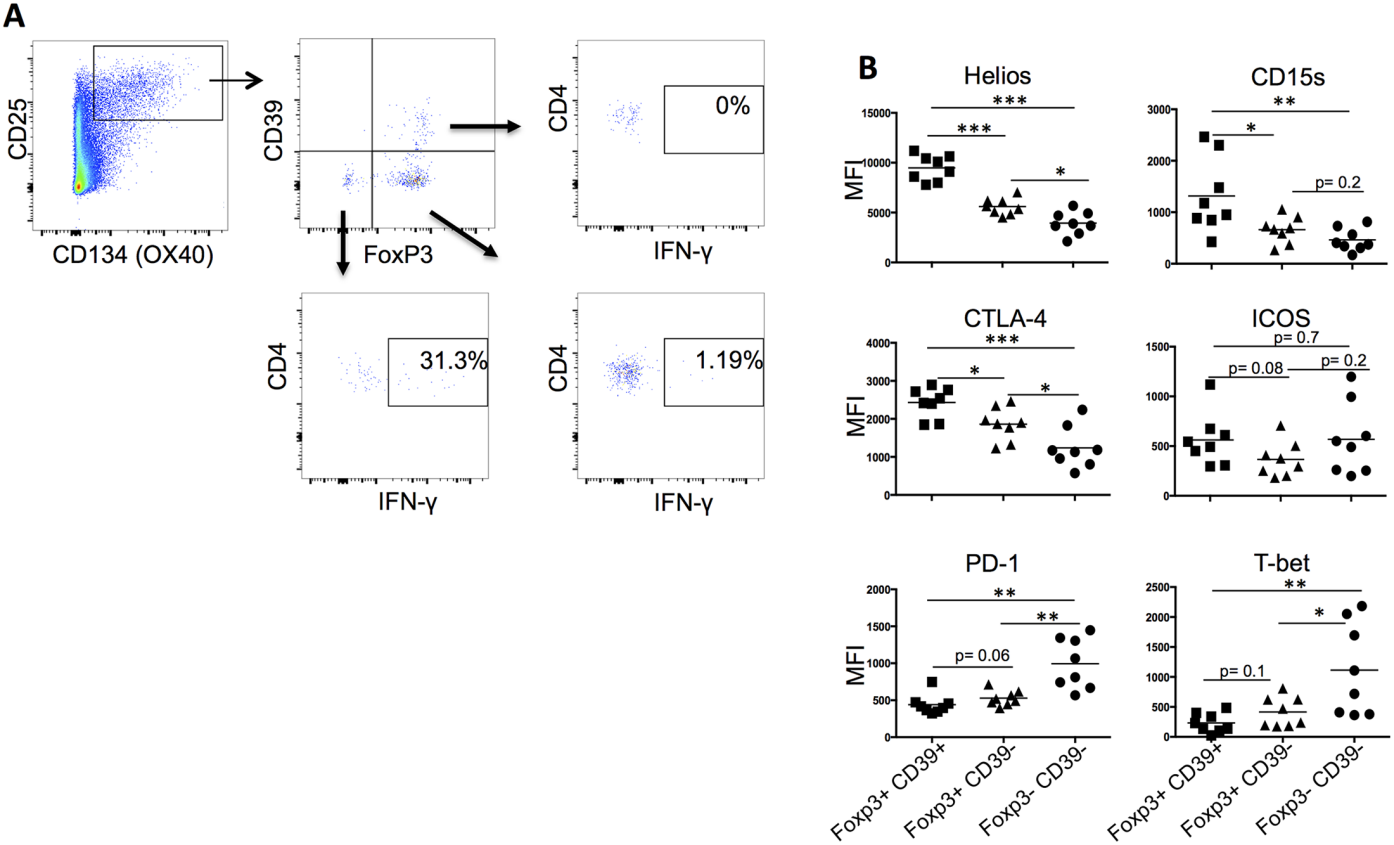
CD25+CD134+ CD39+FoxP3+ cells have bona fide Tregs phenotype. (A) Gating strategy used for detecting CMV-specific CD4+CD25+CD134+ CD39+FoxP3+ Tregs, and IFN-γ producing CD25+CD134+CD39-FoxP3- and CD25+CD134+ CD39-FoxP3+. PBMCs from CMV+ individuals were stimulated with CMV lysate for 44hrs. Flow cytometry was used to detect CMV-specific cells as described in the methods. (B) Mean fluorescence intensity (MFI) of Helios, CD15s, CTLA-4, ICOS, PD-1 and T-bet molecules were measured by flow cytometry in 8 CMV+ individuals to detect CMV-specific subsets (CD134+CD25+Foxp3+CD39+, CD134+CD25+Foxp3+CD39- and CD134+CD25+Foxp3-CD39-). P values were considered significant when < 0.05.

To describe more thoroughly the phenotype of these cells, we assessed the expression of several putative Tregs markers, such as Helios, CTLA-4 and CD15s, which has recently been reported as a novel marker of highly suppressive Tregs [35]. Our results show that these markers were more abundantly expressed on CD39^+^FoxP3^+^CD25^+^CD134^+^ in comparison to both CD39^-^FoxP3^+^ and CD39^-^FoxP3^-^ CD25^+^CD134+ cells as represented by the mean fluorescence intensity (MFI) for each molecule (Fig 8B). ICOS expression was similar in all subsets but T-bet and PD-1 expressions were higher in CD39^-^FoxP3^-^ as compared to the other subsets. Similar data were obtained with HIV-specific CD39^+^FoxP3^+^ cells from patients, with CTLA-4 being significantly higher on these cells (S2 Fig). Of note, MFI of CTLA-4, Helios, CD15s molecules remained stable on HIV-specific Tregs at wk0, wk16 and wk36 (S2 Fig). In addition to the phenotype, we have also assessed the suppressive capacity of CD39^+^Tregs by measuring cytokine production (TNF-α or IFN-γ) by flow cytometry (using the OX40 assay) and by ELISpot, before and after CD39^+^CD4^+^ cell depletion. In S3 Fig we show a representative flow cytometry experiment (1 out of 5 that have been performed on 3 HIV^+^ and 2 CMV^+^ individuals) where total PBMCs or CD39^+^CD4^+^-depleted PBMCs have been stimulated by either CMV, gag-p24 or SEB. As indicated in the figure, the frequency of total CD134^+^CD25^+^CD4^+^ CMV-specific cells decreased from 3.62% to 1.44% (before and after depletion, respectively) and within this subset, there was a significant decrease in CD39^+^Foxp3^+^ cells (30.3% vs 3.66% before and after depletion respectively), which demonstrates depletion efficacy (S3 Fig). Importantly, we observed an increase of about 25–30% in TNF-α production (p<0.05) after CD39^+^CD4^+^ depletion in CD134^+^CD25^+^CD4^+^ CMV- (mean± SEM, 6.55± 0.44% vs 9.92± 2.78%) and HIV- (mean± SEM, 6.2± 3% vs 8.1± 3.7%) specific cells, demonstrating that CD39^+^Tregs have a suppressive function and are able to inhibit cytokine production. Similar trends were obtained when we performed IFN-γ ELISpot experiments using total PBMCs and CD39+CD4+-depleted PBMCs stimulated with p24 15-mer peptide pool (S3 Fig).

Altogether, these data indicate that antigen-specific CD39^+^FoxP3^+^CD25^+^CD134^+^ cells display bona fide Tregs characteristics.

Globally, these results underline the negative impact of HIV-specific Tregs on HIV-specific effector responses and on the control of HIV replication and reveal the effect of IL-2 decreasing a population of HIV-specific Tregs expressing CD39.

At the same time, we observed after IL-2 therapy a trend for an increase in CD39^-^FoxP3^-^CD25^+^CD134^+^ effector cell frequency at wk36 compared to wk0 (Fig 6F). Moreover, an inversed correlation with VL was observed but was not statistically significant (Fig 6G). In contrast, the fraction of CD25^+^CD134+ HIV-specific cells T cells expressing FoxP3 but not CD39 (CD39^-^FoxP3^+^CD25^+^CD134^+^) increased at wk36 (p<0.05; Fig 6D) and showed an inverse correlation with VL (r = -0.7 and p = 0.05; Fig 6E).

Contrary to CD45RO^-^CD25^+^CD127^low^FoxP3^+^ Tregs, CD39^+^FoxP3^+^CD25^+^CD134^+^ HIV-specific Tregs did not show an inverse correlation with CD95^+^PD1^+^ CD4^+^ T cells (S4 Fig) suggesting that they do not impact on T-cell exhaustion.

## Discussion

Lessons from the previous phase III trials (SILCAAT and ESPRIT) that have been conducted to expand the CD4^+^ T-cell pool in HIV-1-infected patients and yielded no clinical benefit [26], were very disappointing which somehow banished the use of rIL-2 in HIV immunotherapy and made the medical community increasingly reluctant to use recombinant cytokines in this context. Greater improvements were seen however, in the ANRS 093 study where patients were randomized to continue either HAART alone or to receive therapeutic vaccination followed by 3 cycles of subcutaneous IL-2 [31].

In this study, we revisited the role of IL-2 in immunotherapy and investigated the dynamics of Tregs subsets in HIV-infected patients receiving therapeutic vaccination combined to IL-2 therapy. We show that while peripheral global CD25^+^CD127^low^FoxP3^+^ Tregs significantly increased at wk36 after IL-2 therapy, HIV-specific CD39^+^FoxP3^+^ Tregs decreased. Importantly, we show that after IL-2 therapy, there was a significant decrease in HIV-specific CD134^+^CD25^+^CD39^+^FoxP3^+^ Tregs in contrast to IFN-γ-producing CD39^-^FoxP3^+^ and CD39^-^FoxP3^-^ HIV-specific subsets. Why CD39^-^ but not CD39^+^ cells were preferentially expanded by IL-2 treatment remains to be clarified. Interestingly, HIV-specific CD39^+^FoxP3^+^ Tregs were inversely correlated with viral load after treatment interruption, which is reminiscent of our recent report [9]. Moreover, we observed a significant decrease in T-cell exhaustion as demonstrated by the down-regulation of PD-1, Tim-3, 2B4 and Blimp-1 expressions, which inversely correlated with total memory CD25^+^CD127^low^FoxP3^+^ Tregs but not with HIV-specific CD39^+^FoxP3^+^ Tregs. These results make IL-2 a promising cytokine to be reconsidered for use as an adjuvant in the context of chronic infections as long as it is used in combination with a vaccine. Moreover, timing and dosing of IL-2 need to be carefully considered for each situation, as previous studies showed that exogenous IL-2 is detrimental during the expansion phase after acute LCMV infection for both CD8 and CD4 virus-specific T cells [36]. Of note, excessive IL-2 during T-cell expansion modulates T-cell differentiation towards terminal effectors, which increases cell death and hampers memory formation, whereas IL-2 administration during contraction led to increased cell survival [36]. Therefore, a careful examination of IL-2 dosing and timing as a means to shift the balance between immunity and tolerance could be the key for future studies.

The benefit in using systemic low-dose IL-2 has been reported in several autoimmune disorders [15–21]. Studies indicated that this approach is well tolerated and the advantageous function of low-dose IL-2 has been linked to the expansion of Tregs in controlling immune responses and establishing tolerance [22]. However, several studies reported that Tregs have an ambiguous role in HIV infection, as they decrease immune activation, which is beneficial for HIV-infected individuals, but they also suppress anti-HIV responses, which is undesirable in such condition (reviewed [1]). Part of this ambiguity is related to Tregs identification and quantification (reviewed in [37]. In addition, there is another important aspect that has never been taken into account and that is the simultaneous examination of the global picture and dynamics of the different Tregs subsets in the same patient. These subsets include HIV-specific, naïve and activated/memory circulating global Tregs. In this study we have delineated the dynamics of Tregs subsets after vaccination and IL-2 therapy, and according to previous studies (SILCAAT and ESPRIT phase III trials) [26,27,38], we found that Tregs frequencies significantly increased. We demonstrated that naïve CD45RO^-^CD25^+^CD127^low^FoxP3^+^ cells were the most affected by IL-2 treatment as compared to the total memory counterpart. Importantly, we revealed a significant decrease in CD25^+^CD127^low^FoxP3^+^CD39^+^ Tregs, which were shown to be highly suppressive [6,34]. More interestingly, when we analysed HIV-specific CD39^+^FoxP3^+^ Tregs, we found that they decreased significantly after IL-2 therapy compared to HIV-specific CD39^-^FoxP3^+^ and CD39^-^FoxP3^-^ effectors. It is important to point-out that HIV-specific CD4^+^CD134^+^CD25^+^ CD39^+^FoxP3^+^ Tregs correlated positively with viral load after treatment interruption, which suggests that Tregs may represent a potential reservoir for the virus. Indeed, there is strong evidence that Tregs can become latently infected and may represent a potentially important HIV reservoir as i) they expand in blood and tissues in chronically HIV-infected patients and SIV-infected macaques [39]; ii) HIV/SIV DNA levels in Tregs from HIV-infected patients on HAART and SIV-infected rhesus macaques are higher than that of non-Tregs [40,41]; and iii) Tregs are less susceptible to cell death than conventional T cells [39]. As such, therapeutic interventions aiming at Tregs depletion may directly contribute to the reduction of the size of virus reservoir [42]. In addition, IL-2 signaling through CD25 facilitates HIV replication *in vitro* and facilitates homeostatic proliferation of CD25^+^FoxP3^+^CD4^+^ Tregs [39]. Thus the implication of HIV-specific CD39^+^FoxP3^+^ to the establishment of HIV reservoir needs to be clarified and is currently under investigation.

In contrast to HIV-specific CD39^+^FoxP3^+^, both HIV-specific CD39^-^FoxP3^+^ and CD39^-^FoxP3^-^ non-Tregs, which are enriched in IFN-γ producing effectors, inversely correlated with viral load after treatment interruption. These data are in line with previous LCMV mouse studies, where daily therapeutic low-dose IL-2 increased virus-specific T-cell responses, thus resulting in decreased viral burden [43].

There must be a slow turnover of circulating Tregs that are constantly dividing as their absolute number remains constant [44]. It needs to be clarified whether their survival and function relies solely on IL-2. One possible explanation to our results showing a significant increase in global Tregs compared to HIV-specific Tregs after vaccination and IL-2 therapy could be the fact that the former can be exclusively dependent on IL-2 for expansion, survival and function, whereas the latter are largely controlled by signaling through the TCR for their generation. This could explain their role in controlling HIV-specific responses without relying that much on IL-2 for their function. Indeed, these hypotheses need to be verified in our future investigations.

We have previously shown in Dalia1 clinical study [45] that CD4^+^CD25^+^CD134^+^ HIV-specific responses induced after vaccination, inversely correlated with viral load rebound after treatment interruption [9]. Similar outcomes were found here in ANRS 093 study, where we measured CD4^+^ HIV-specific responses at wk0, wk16 and wk36 using the “OX40 assay”. In the primary ANRS 093 study [30], we have reported that HIV-specific immune responses at wk0, before therapeutic vaccination and IL-2 administration, did not correlate with the control of viral replication following TI in contrast to wk16 responses. In the present study we extend these data, and support the role of IL-2 in boosting pre-existing HIV-specific effector T cells and suggest its use as an adjuvant in combination with an efficient HIV-vaccine.

Another positive outcome in this ANRS 093 study is the significant decrease in CD4^+^ and CD8^+^ CD95^+^PD-1^+^ frequencies as well as in Tim-3, Blimp-1 and expressions at wk36 post IL-2 therapy. This expression of CD95^+^PD-1^+^ on CD4 T cells was inversely correlated with total memory CD25^+^CD127^low^FoxP3^+^ Tregs. Our results are in accordance with previous data in chronic LCMV infection where IL-2 treatment was associated with decrease expression of inhibitory receptors consistent with a less exhausted phenotype [43]. We believe that our results shed light on the contrasting impact of IL-2, as on one hand we observed an increase in global CD25^+^CD127^low^FoxP3^+^ Tregs which might impact on T-cell exhaustion, and on another hand a decrease in HIV-specific CD134^+^CD25^+^CD39^+^FoxP3^+^ Tregs, that positively correlated with viral load confirming the non-beneficial role of HIV-specific Tregs in the context of therapeutic vaccination [9]. Altogether, these data uncover some benefits in the use of IL-2 as an adjuvant in HIV-vaccines protocols.

Given that therapeutic vaccines and cytokines have been commonly used to enhance HIV-specific cell-mediated immune responses and to suppress virus replication, revisiting IL-2 for its use as an adjuvant in HIV therapeutic vaccination, appears timely. While the former is important to stimulate HIV-specific T-cell responses, the latter may support the expansion of the stimulated virus-specific T cells. The recent success using checkpoints inhibitors in the cancer field [46], have started to be introduced in preclinical models of HIV with the objective to preserve the function of HIV-specific CD8^+^ T cells from exhaustion and target directly HIV cell reservoir [47–49]. These major advances in immunological intervention strategies provide a rationale for revisiting the use of IL-2 together with immune checkpoint molecules in combinatorial therapeutic strategies to enhance the vaccine-elicited immune response and thus gain more efficient control of virus replication (review in [50]).

Studies using the LCMV model indicated that combined IL-2 and PD-1 blockade therapy represents a promising therapy for increasing CD8 T cell function and reducing viral loads during chronic infection [43]. Additional studies will need to be performed to determine whether IL-2 or combined IL-2 therapy helps “reprogram” exhausted CD8 T cells in humans.

Altogether our data highlight the positive role of IL-2 therapy in HIV infection, as despite an increase in CD25^+^CD127^low^ Tregs, HIV-specific effector CD4^+^ T cells were greatly increased and were able to reduce viral load. This emphasizes the distinct roles that IL-2 therapy can have during an autoimmune manifestation where low-dose IL-2 therapy can increase Tregs, resulting in clinical improvement (reviewed in [22]) and during chronic infection where virus-specific T cells can expand and impact on viral load. Thus combining IL-2 therapy with vaccination seem to be promising and useful in clinical strategy for reversing T cell exhaustion during chronic infections and boosting antigen-specific effector responses leading to an enhanced reduction in viral burden.

## Materials and methods

### Patients, vaccines and study design

Seventy patients over 18 years, with asymptomatic HIV-1 infection and CD4 T-cell counts > 350 cells/ml and plasma HIV RNA < 50 copies/ml and who have been previously treated with HAART for at least 1 year were eligible. Patients who received subcutaneous IL-2 in a previous study [31], were also eligible and the last cycle was administered at least 3 months prior study entry. Patients were randomized to continue either HAART alone (n = 37; control group) or combined with 10^6.6^ median infectious dose (ID50) ALVAC vCP 1433 and 3 mg HIV-LIPO-6T (both given by Aventis Pasteur) administered intramuscularly at weeks 0, 4, 8, 12 followed by three cycles of subcutaneous IL-2 (given by Chiron Europe) at weeks 16, 24, 32 (4.5 MIU, twice daily for 5 days) (n = 33; vaccine group) (Fig 1a). ALVAC-HIV expresses several HIV genes: for gp120 (MN strain) and a part of the anchoring transmembrane region of gp41 (LAI strain); for the p55 polyprotein, expressed by gag (LAI strain); for a portion of pol encoding the protease; and for genes expressing cytotoxic T lymphocyte peptides from pol and nef. The HIV-LIPO-6T vaccine is a mixture of the tetanus toxoid TT-830-843 class II-restricted universal CD4 epitope combined with five peptides: Gag 17–35, Gag 253–284, Nef 66–97, Nef 116–145 and Pol 325–355 from HIV-1 LAI. Patients with HIV RNA < 50 copies/ml stopped HAART at week 40. Following treatment interruption, HAART was reinitiated if HIV RNA was > 50 000 cp/ml at 4 weeks after interruption or > 10 000 copies/ml at 8 weeks after interruption or thereafter at any protocol follow-up visit.

### Blood samples

All experiments were performed on freshly thawed cells that were left to rest for 5–6 hours in human serum-supplemented medium at 37°C.

### Flow cytometry staining and phenotyping

All staining experiments were performed at 4°C for 30 minutes. Antibodies used were CD3-PerCPCy5.5, CD3-AF700, CD4-Brilliant Violet 605, CD8-APC-Cy7, CD25-APC, CD134-PE, OX40-APC, TNF-α-PECy7, CD154-APC, CD152-PCF594, Tbet-PerCPCy5.5, PD1-PeCy7, CD15s-Brilliant Violet 421, Helios-PE, ICOS-PeCy7, CD39-Brilliant Violet 711 (Becton Dickinson (BD) Biosciences), CD4-Alexa Fluor 700, IFN-γ-eFluor450, IL2-PerCPeFluor710, Streptavidin-Alexa Fluor 700 (eBioscience), FoxP3-Alexa Fluor 488, CD25-Brilliant Violet 421 (BioLegend), CD39-biotin, CD127-PE, CD4-APCVio770, CD3-APCVio770 (Miltenyi biotec), Streptavidin-ECD, CD45RO-ECD (Beckman Coulter). LIVE/DEAD fixable aqua staining kit (Life technologies) was used to discriminate live and dead cells. For intracellular staining, FoxP3 buffer set (eBioscience) was used. Cell acquisition was performed by an LSR II (Becton Dickinson) and analyses were performed using FlowJo software.

### T-cell functional assays

The “OX40 assay” is described in details elsewhere [9,51]. Briefly, two million PBMCs or Tregs-depleted cells were plated in 24-well plate and stimulated with 1μg/mL CMV lysate (Behring) or 2μg/mL of HIV-1 Gag p24 peptide pool for 44 hours. In the last 6 hours, 1μg/mL of Brefeldin A (Sigma) was added to block the secretion of IFN-γ, IL-2 and TNF-α. Cells were then collected and stained for subsequent analysis by flow cytometry (BD LSR II).

In some experiments CD39^+^CD4^+^ cells were depleted from PBMCs before performing the “OX40 assay”. Briefly, we first isolated CD4+ T cells that were negatively selected using CD4 Microbeads (Miltenyi Biotec). The non-CD4 cell fraction has been collected too and put in a 12 well-plate for the rest of the experiment. On the purified CD4+ cells, biotinylated anti-CD39 mAb was added. After incubation and several washes, CD39-labelled CD4^+^ T cells were incubated with anti-biotin Microbeads (Miltenyi Biotec) to positively collect the CD39^+^CD4^+^ fraction. The non-CD39 fraction has been collected too and added to the well together with the non-CD4 to “reconstitute” the PBMCs (CD39^+^CD4^+^-depleted PBMCs). Hence, “OX40 assay” was then performed as described above on total PBMCs and on CD39+CD4+-depleted PBMCs.

### EliSpot ELISpot assay

Evaluation of HIV-specific cells producing interferon-γ (IFN-γ) was assessed on frozen total PBMCs and CD39^+^CD4^+^-depleted PBMCs (obtained as described above) and using an ELISPOT assay performed as described [31]. Antigens included 9 or 18 pools of 15-mer peptides covering HIV-1 Gag (11 pools), reverse transcriptase (four pools) and Nef (three pools) (Neosystem) at 2 μg/ml. The IFN-γ-producing cells were counted using an automated microscope (Zeiss, Le Pecq, France), expressed as spot-forming cells (SFC)/10^6^ PBMC and averaged over triplicate wells. The number of specific SFC/10^6^ PBMC was calculated by subtracting the negative control value (unstimulated cells) from the established SFC count. Positive responses were defined as greater than 100 SFC/10^6^ PBMC over background and at least twofold the background value.

The breadth of the response was the number of recognized pools among 18 pools tested and the magnitude was defined as the sum of positive responses to individual pools (total SFC/10^6^ PBMC).

### Statistical analysis

Analyses of differences between pre- and post-vaccination time points were done by Wilcoxon matched-pairs signed rank test. Correlations were assessed by Spearman correlation coefficients.

Prism 5.0, version 5.0d, (GraphPad Software, Inc.) was used for statistical analyses. P values were considered significant when < 0.05.

### Ethics statement

Ethics committee (CCPPRB Créteil Henri Mondor) approved this study and written informed consent from all patients, in accordance with the Declaration of Helsinki, were obtained prior to study initiation.

All blood samples used in the study were obtained from ANRS (Agence Nationale de la Recherche sur le SIDA) and were anonymized.

## Supporting information

S1 Fig Mean fluorescence intensity (MFI) of Blimp-1 (A), Tim-3 (B), CD38 (C) and HLA-DR (D) molecules were measured by flow cytometry in CD4+CD95+ cells from HIV-infected patients at wk0, wk16 and wk36. (TIFF)

S2 Fig Mean fluorescence intensity (MFI) of CTLA-4 was measured by flow cytometry in CD4^+^CD25^+^CD134^+^CD39^+^FoxP3^+^, CD4^+^CD25^+^CD134^+^ CD39^-^FoxP3^+^ and CD4^+^CD25^+^CD134^+^ CD39^-^FoxP3^-^ subsets from an HIV-infected patient at wk0, wk16 and wk36. P values were considered significant when < 0.05.
(TIFF)

S3 Fig Cytokine expression before and after CD39^+^Tregs depletion. (A, top and bottom panels) Representative flow cytometry experiment (1 out of 5) using « OX40 assay » to measure the frequency of CD134^+^CD25^+^CD4^+^ and CD134^+^CD25^+^CD4^+^ Foxp3^+^CD39^+^ cells after stimulation of total PBMCs or CD39^+^CD4^+^-depleted PBMCs with CMV lysate. (B) TNF-α production in CD134^+^CD25^+^ CMV-specific cells before and after CD39^+^CD4^+^ Tregs depletion. (C) TNF-α production in CD134^+^CD25^+^CD4^+^ after stimulation with CMV lysate in CMV+ individuals (n = 2) or with Gag p24 peptide pool in HIV+ patients (n = 3). SEB was used as a positive control. (D) IFN-γ ELISpot experiments using total PBMCs and CD39^+^CD4^+^-depleted PBMCs (n = 2 HIV-infected patients) stimulated with p24 15-mer peptide pool (SFC are expressed per 10^6^ cells). Prism 5.0, version 5.0d, (GraphPad Software, Inc.) was used for statistical analyses. P values were considered significant when < 0.05. Standard Error of the mean (SEM) are represented for histograms shown in C and D.
(TIFF)

S4 Fig Correlation of CD25^+^CD134^+^CD39^+^FoxP3^+^ HIV-specific Tregs and CD4^+^CD95^+^PD1^+^ at wk36. Correlations were calculated using spearman correlation coefficients (Prism 5.0, version 5.0d). P values were considered significant when < 0.05.
(TIFF)
