# Supplementary figures and images for: Negative modulation of suppressive HIV-specific regulatory T cells by IL-2 adjuvanted therapeutic vaccine
Source: PLoS Pathog. 2017 Jul 14;13(7):e1006489. doi: 10.1371/journal.ppat.1006489 (PMC5529021; doi:10.1371/journal.ppat.1006489)

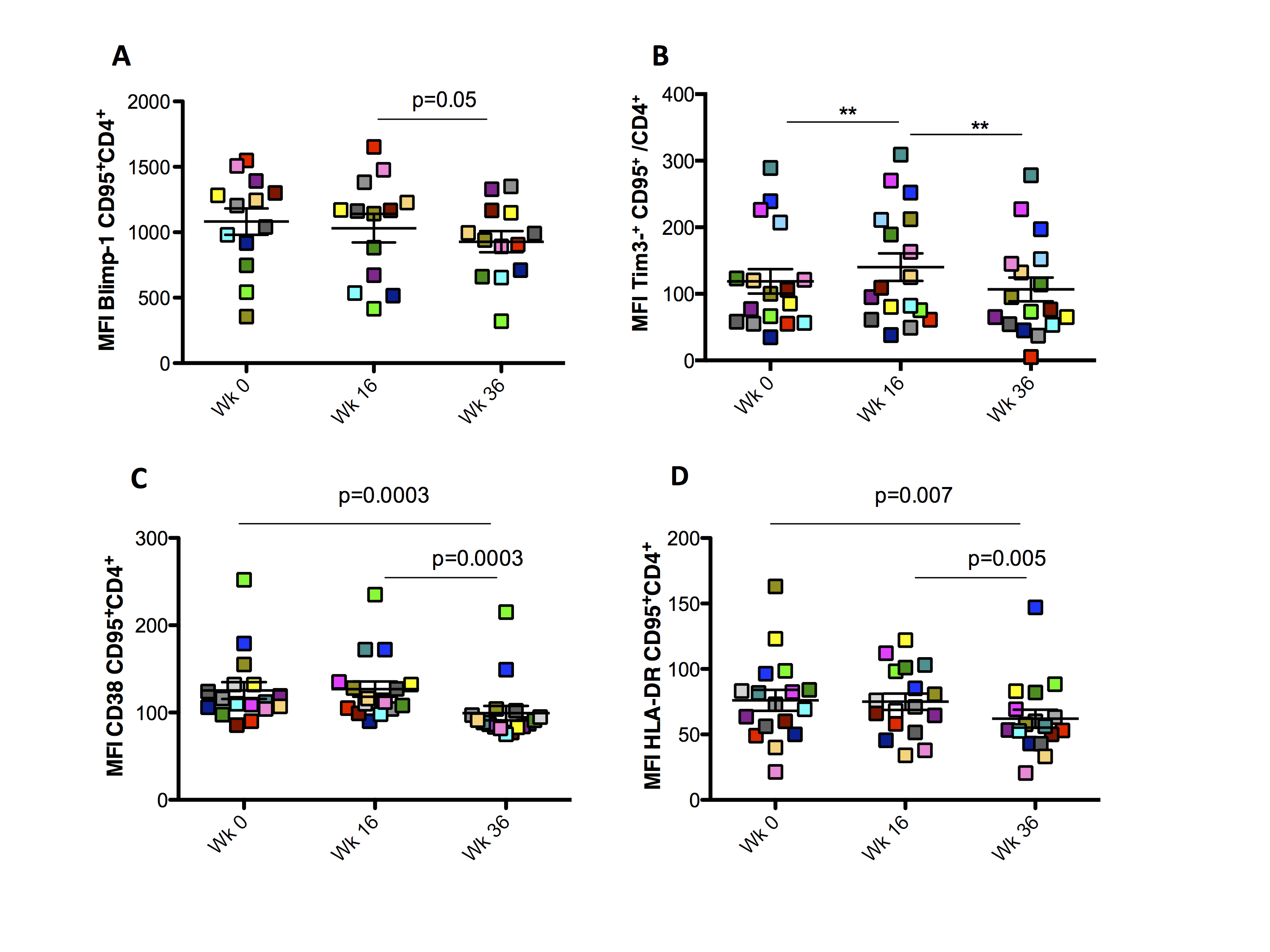

Supplement: S1 Fig — (TIFF) [file ppat.1006489.s001.tiff]

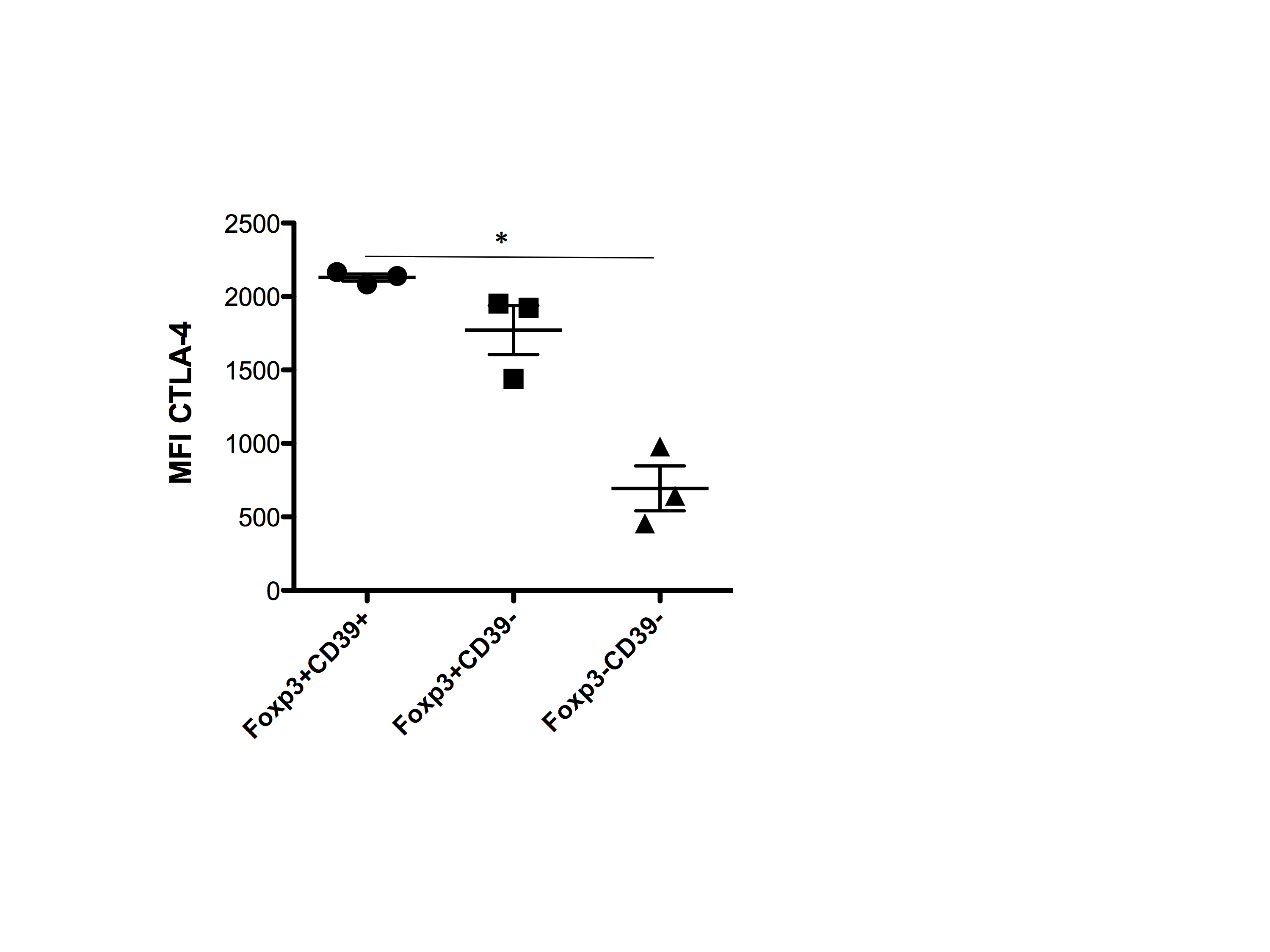

Supplement: S2 Fig — P values were considered significant when < 0.05. (TIFF) [file ppat.1006489.s002.tiff]

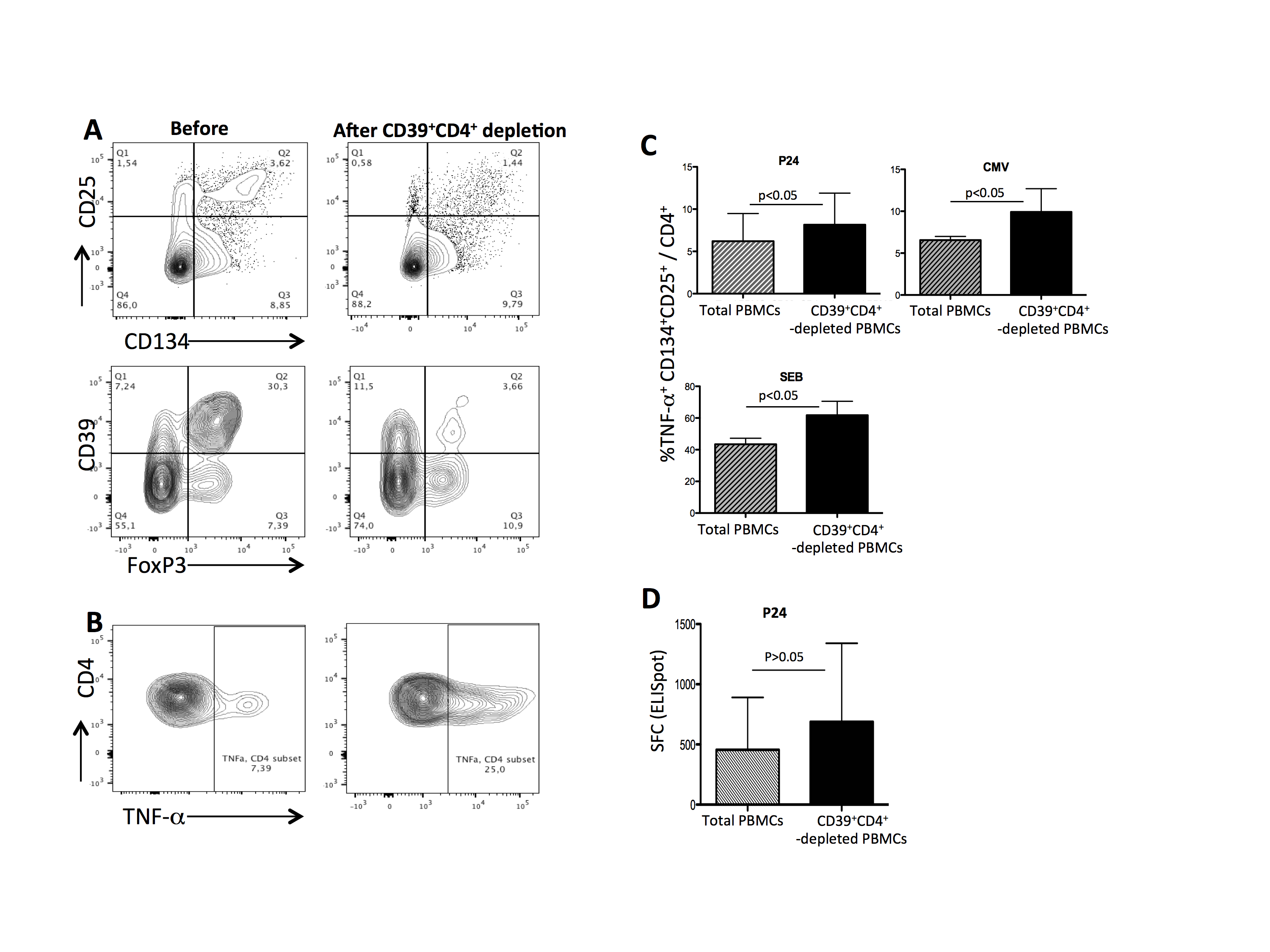

Supplement: S3 Fig — (A, top and bottom panels) Representative flow cytometry experiment (1 out of 5) using « OX40 assay » to measure the frequency of CD134+CD25+CD4+ and CD134+CD25+CD4+ Foxp3+CD39+ cells after stimulation of total PBMCs or CD39+CD4+-depleted PBMCs with CMV lysate. (B) TNF-α production in CD134+CD25+ CMV-specific cells before and after CD39+CD4+ Tregs depletion. (C) TNF-α production in CD134+CD25+CD4+ after stimulation with CMV lysate in CMV+ individuals (n = 2) or with Gag p24 peptide pool in HIV+ patients (n = 3). SEB was used as a positive control. (D) IFN-γ ELISpot experiments using total PBMCs and CD39+CD4+-depleted PBMCs (n = 2 HIV-infected patients) stimulated with p24 15-mer peptide pool (SFC are expressed per 106 cells). Prism 5.0, version 5.0d, (GraphPad Software, Inc.) was used for statistical analyses. P values were considered significant when < 0.05. Standard Error of the mean (SEM) are represented for histograms shown in C and D. (TIFF) [file ppat.1006489.s003.tiff]

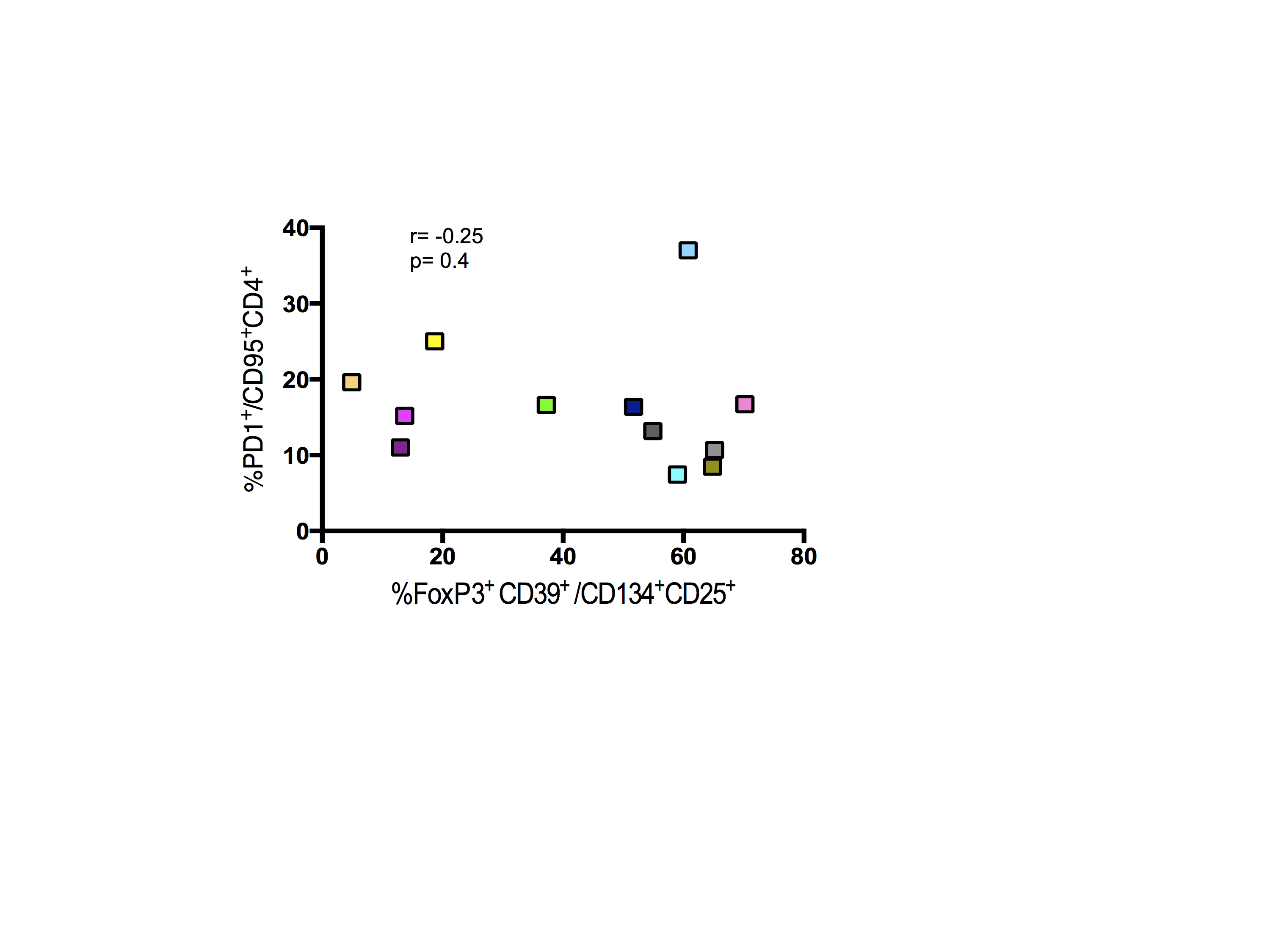

Supplement: S4 Fig — Correlations were calculated using spearman correlation coefficients (Prism 5.0, version 5.0d). P values were considered significant when < 0.05. (TIFF) [file ppat.1006489.s004.tiff]
